# Monitoring of Low-Intensity Exposures via Luminescent Bioassays of Different Complexity: Cells, Enzyme Reactions, and Fluorescent Proteins

**DOI:** 10.3390/ijms20184451

**Published:** 2019-09-10

**Authors:** Nadezhda S. Kudryasheva, Ekaterina S. Kovel

**Affiliations:** 1 Institute of Biophysics, Federal Research Center “Krasnoyarsk Science Center, Russian Academy of Sciences, Siberian Branch“, Krasnoyarsk 660036, Russia; 2 Siberian Federal University, Krasnoyarsk 660041, Russia; 3 Institute of Physics, Federal Research Center “Krasnoyarsk Science Center, Russian Academy of Sciences, Siberian Branch“, Krasnoyarsk 660036, Russia

**Keywords:** luminescence bioassays, bacterial cells, enzymes, fluorescent protein, low-intensity factors, hormesis, radiation, bioactive compounds, antioxidant activity

## Abstract

The current paper reviews the applications of luminescence bioassays for monitoring the results of low-intensity exposures which produce a stimulative effect. The impacts of radioactivity of different types (alpha, beta, and gamma) and bioactive compounds (humic substances and fullerenols) are under consideration. Bioassays based on luminous marine bacteria, their enzymes, and fluorescent coelenteramide-containing proteins were used to compare the results of the low-intensity exposures at the cellular, biochemical, and physicochemical levels, respectively. High rates of luminescence response can provide (1) a proper number of experimental results under comparable conditions and, therefore, proper statistical processing, with this being highly important for “noisy” low-intensity exposures; and (2) non-genetic, i.e., biochemical and physicochemical mechanisms of cellular response for short-term exposures. The results of cellular exposures were discussed in terms of the hormesis concept, which implies low-dose stimulation and high-dose inhibition of physiological functions. Dependencies of the luminescence response on the exposure time or intensity (radionuclide concentration/gamma radiation dose rate, concentration of the bioactive compounds) were analyzed and compared for bioassays of different organization levels.

## 1. Introduction

**Figure 1:**
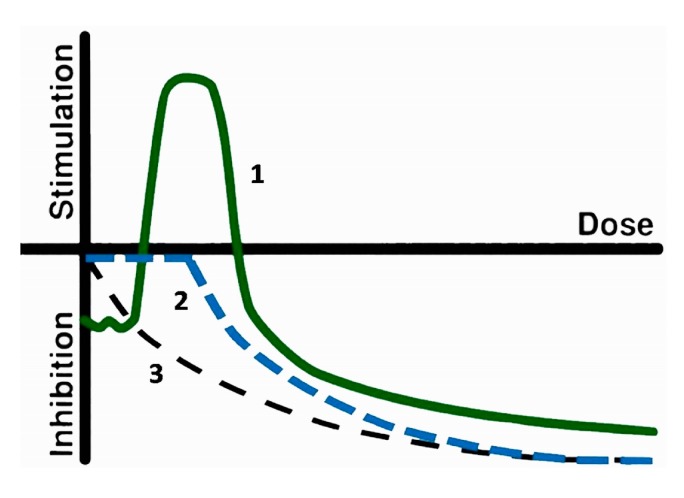
Scheme of dose–effect models: 1, hormesis; 2, threshold; 3, linear.

All biological objects on the Earth are exposed to low-intensity factors—radioactive, chemical, and electromagnetic. Currently, the accumulation of evidence on the inhibitory and activating effects of these factors is ongoing. The activation of the physiological functions of organisms is associated with the term “hormesis”, which implies a favorable biological response to the low impact of toxins and other stressors. This term was introduced by Southam and Ehrlich in 1943 [1,2]. Over the past decades, an exponential increase in citation for hormesis has been observed in the biomedical community [2,3,4,5]. The hormesis model is based on the non-linear dependence of an effect on a dose of toxic compounds, as shown schematically in Figure 1. Hormesis is considered a basic model; two other models (linear and threshold) can be considered as particular cases of hormesis [2,5].

There exists evidence that hormesis is a highly generalized phenomenon; it does not depend on the level of biological organization (cells, organs, or organisms).

Understanding and predicting the responses of organisms to low-intensity exposures is of high importance for practical applications. This understanding should be based on the molecular mechanisms of these effects, which are intensively studied now [1,2,3,4,5,6] but not yet clear. We suppose that simple bioassay systems, such as cells and enzymes, will allow understanding of the low-intensity effects at cellular and biochemical levels, respectively. The next stage of the investigation should be concerned with the level of physicochemical processes (energy, electron, or hydrogen transfer) and application of the simplest bioassay systems.

The current review considers an application of the luminescence bioassay systems of different organization levels (cells, enzyme reactions, and fluorescent proteins) for studying the mechanisms (cellular, biochemical, and physicochemical, respectively) of low-intensity exposures.

It is known that bioassays form a basis for toxicological investigations. The term “toxicity” is of biological origin; it means a suppression of an organism’s physiological functions [7]. Bioassays have been used to monitor toxicity for centuries. Classic bioassays use mice, frogs, fish, crustaceans, plants, microorganisms, and others [8,9,10,11]. Their physiological functions monitored in the bioassay procedure are respiration, lifetime, rates of growth or movement, and the bioluminescence intensity. The main feature of all bioassays is an integral response. This means that a lot of processes of different organization levels (cellular, biochemical, physicochemical) contribute non-additively to the physiological functions of organisms, changing their growth, respiration, lifetime, movement, bioluminescent intensity, etc. This is a basis for nonspecificity of biological analyses. Bioassays provide a non-specific monitoring of environments and are widely used in toxicological investigations. Traditionally, bioassays are applied as “signal” tests, and their results point to the necessity for more detailed and expensive analyses, chemical or biochemical ones. The detailed characteristics of biological assays and their correlations with chemical analyses are discussed in [12].

The luminescence feature of the bioassays provides a proper registration of biological responses. Luminescence intensity is a physiological test parameter monitored in the course of the bioassay procedure. The advantages of luminescence registration are high rates of analysis (down to 1–3 min), ease of use, high sensitivity, and availability of instruments and reagents [13,14,15]. Since the luminescent type of registration is not time consuming, it can provide a lot of experimental results under comparable conditions and, hence, proper statistical processing. This advantage is important as biological analyses are usually characterized by lower reproducibility than chemical or radiometric assays. In addition, this advantage is of particular importance for low-intensity exposures, which can be described, as a rule, in terms of “stochastic effects” [6]. Besides, the quick response can contribute to the investigation of the non-genetic mechanisms of low-intensity exposures.

The most well-known luminescent cellular bioassay is based on luminous marine bacteria; the main test parameter here is bioluminescence intensity. Bacterial bioluminescence is sensitive to toxic compounds; this is a reason why the marine bacterium has been used for several decades to assess environmental toxicity [13,14,15,16,17,18,19].

Glowing of marine bacteria is based on bioluminescence reactions, i.e., chemiluminescence reactions catalyzed by specific enzymes, bacterial luciferases. In these reactions, molecular oxygen oxidizes a long-chain aldehyde and flavin mononucleotide (FMN) followed by the electron-excited flavin derivative formation and light emission. From the early 1990s, bacterial bioluminescent reactions have been applied as a bioassay system for toxicity monitoring [20,21,22,23]. This system is one of the most widely used enzymatic bioassays.

Solid immobilized enzymatic and bacterial preparations are being developed now as a basis for bioluminescent biosensors [20,23,24,25,26]. The effects of toxic compounds on the bioluminescence enzymatic system were classified in [27]; the classification was developed later in [28,29,30,31]. This classification describes (1) physicochemical, (2) chemical, and (3) biochemical processes in the bioluminescence assay systems during exposure to toxic compounds.

Biochemical and physicochemical approaches contribute to the non-genetic aspect of toxic and adaptive effects. These approaches are applicable in frames of the novel “exposome” concept, where the “exposome complements the genome and encompasses the totality of environmental non-genetic exposures” [32,33,34]. The exposome concept originated as a challenge in molecular epidemiology [35] and is concerned with human exposures. Application of simple model organisms and biochemical systems might provide human exposure sciences with fundamental support based on molecular, physicochemical, biochemical, and cellular investigations.

This review analyzes the results of applying the simplest luminescence bioassays (luminous marine bacteria, their enzymes, and fluorescent coelenteramide (CLM)-containing proteins) to study (i) the effects of low-dose radiation of the alpha, beta, and gamma types and (ii) the antioxidant effects of bioactive compounds of natural and artificial origination—humic substances (*HS*) and fullerenols (*F*s), respectively.

The next section of the review, Section 2, justifies the application of CLM-containing fluorescent protein as the simplest multicolor bioassay based on physicochemical processes in the protein complex. Section 3 discusses an application of luminescence bioassays in studying low-dose radiation effects. Section 4 presents the low-concentration antioxidant effects of bioactive compounds.

## 2. Coelenteramide-Containing Fluorescent Protein as the Simplest Multicolor Fluorescent Bioassay

The main structural components of fluorescent proteins are a polypeptide and an aromatic fluorophore; the latter is responsible for light emission. The most important representative of the fluorescent proteins is green fluorescent protein (GFP). It was isolated in 1962 from the jellyfish *Aequorea victoria* by American scientist O. Shimomura (Nobel Prize 2009 in chemistry) A series of fluorescent proteins of different color, homologues to GFP, are now known [36,37]. They are widely used in medical and biological research for labeling individual molecules, intracellular structures, living cells, organs, and whole organisms [38,39].

**Figure 2:**
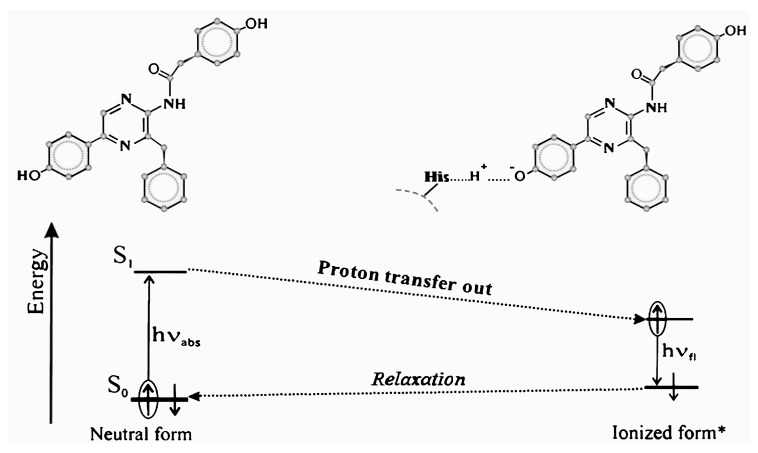
Chemical structure of the coelenteramide (CLM) molecule (neutral and ionized forms) and scheme of the photophysical and photochemical processes in CLM-containing fluorescent proteins.

CLM-containing proteins are the other group of fluorescent proteins. “Discharged aequorin”, a representative of this group, was also isolated and studied by Prof. Shimomura, simultaneously with GFP. He called it blue fluorescent protein (BFP). This group differs from the GFPs in terms of fluorophore formation: the fluorophore of GFP is formed by amino acid chain residuals Ser65-Tyr66-Gly67 as a result of their specific cyclization [40,41], while the fluorophore of BFP is a CLM molecule (Figure 2) which is non-covalently bound inside the hydrophobic cavity of the apoprotein, forming a CLM–apoprotein complex. As opposed to GFPs, the CLM-containing proteins are not widely used, and their potential as color fluorescent biomarkers has not yet been evaluated.

CLM-containing proteins are known to be the products of bioluminescent reactions of marine coelenterates. These reactions are Ca^2+^ dependent, and this is a basis for their biomedical application [42,43]. The biochemical and photophysical mechanisms of the bioluminescence reactions [44,45,46,47,48,49] and spectral characteristics of their products, CLM-containing proteins [50,51,52,53,54,55,56,57], are now under intensive investigation.

**Figure 3:**
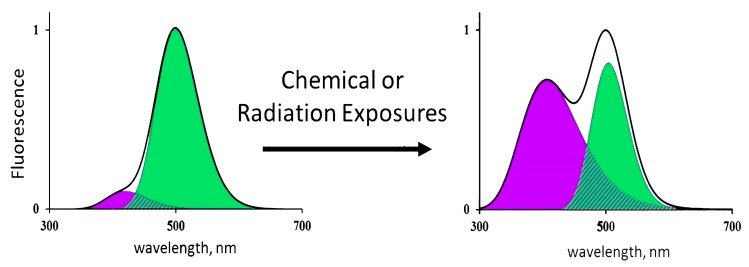
Change of the fluorescence spectra of CLM-containing fluorescence proteins exposed to chemical agents or radiation.

The CLM molecule is a photochemically active compound; its photoexcitation initiates a proton transfer outward (Figure 2). The apoprotein plays the role of a biological catalyzer: amino acid residue His22 is a proton acceptor in this process [45]. The fluorescence colors of the neutral and ionized CLM forms differ (violet and green spectral regions, respectively) (Figure 2 and Figure 3).

The fluorescence characteristics of the neutral and ionized forms of CLM were studied using time-resolved spectroscopy in [53]. Components of the complex photoluminescence spectra of CLM-containing proteins were analyzed in [54].

In general, the photobiochemical process of proton transfer in CLM-containing proteins can be considered an enzymatic bioluminescence reaction of a specific type. Its specificity includes the following: (1) It is a photochemical reaction, but not a chemiluminescent one; it occurs only under UV photoexcitation. Probably, similar processes can be defined as photobioluminescence ones. (2) This photochemical reaction involves a primary physicochemical process of proton transfer. (3) Along with the luminescence of the product of this reaction (ionized CLM form), we observe photoluminescence of the initial substance (neutral CLM form).

Any destructive exposures (radiation, chemical agents, or temperature) can change the structure of the CLM–apoprotein complex, decrease its catalytic activity in the photochemical proton transfer reaction, and change the contributions of the neutral and ionized CLM forms and, hence, the fluorescence color. Figure 3 shows schematically the result of destructive exposures. It is evident that chemical or radioactive impacts decrease the contribution of the neutral (green) component to the overall fluorescence spectrum.

**Figure 4:**
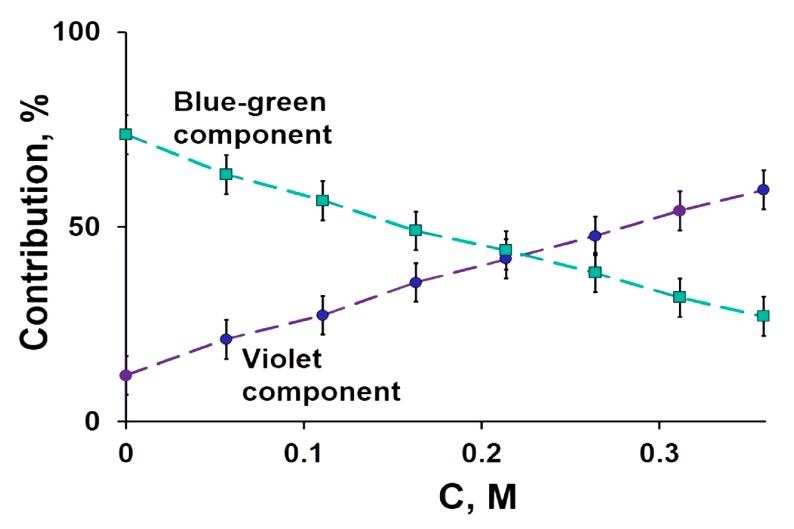
Contributions of spectral components to the fluorescence spectra of CLM-containing protein at different concentrations of glycerol, C.

Figure 4 presents the fluorescence contributions to the spectra of CLM-containing protein with glycerol taken as an example of a chemical agent.

Changes in the contributions of the spectral components were studied earlier under variation of the Ca^2+^ concentration [55], higher temperature [56], exogenous compounds [58,59], and low-dose radiation [60,61].

Therefore, the variation of the fluorescence color of CLM-containing proteins is a result of exposures to destructive factors. We suggested the application of CLM-containing proteins as the simplest toxicity bioassays in [62] based on their ability to change fluorescence color. The toxic effect, in this case, is probably concerned with a decrease in the protein’s catalytic activity in the photochemical processes of proton transfer from CLM to His22 (Figure 2). The bioassay based on CLM-containing protein assumes relations of toxic effects with a primary physicochemical process: proton transfer. Similar to conventional bioassays, the response of the protein to the exposures is “integral”, “non-specific”, and does not depend on the type of exposure (temperature, radiation, or chemical agents).

## 3. Luminescence Bioassays as Tools for Studying Low-Dose Radiation Effects

Study of radiobiological low-dose effects has been intensively developing since the 1970s [63,64,65,66,67], including effects on microorganisms [68,69,70]. The hormesis model is used to describe and explain the activation effects of radiation. The first radiation hormesis tutorial was written by Luckey in 1980 [71].

Luminous marine bacteria have been applied to monitor low-dose radiation effects for about a decade [19,21]. In this period, the effects of alpha- and beta-emitting radionuclides americium-241, uranium 235/338, and tritium, as well as those of gamma radiation, have been investigated [19,21,22,60,61,72]. It was shown that the bacterial bioluminescence response to radionuclides americium-241 and tritium includes three stages: (1) threshold, (2) activation, and (3) inhibition. We have chosen these two radionuclides for presentation here due to their radioecological significance: both radionuclides are accumulated in the environment now. Tritium is a by-product of many radiochemical reactions in the nuclear industry; americium-241 is a by-product of plutonium decay, its radiation lifetime is high – 432.6 years, and it is able to accumulate in living cells.

**Figure 5:**
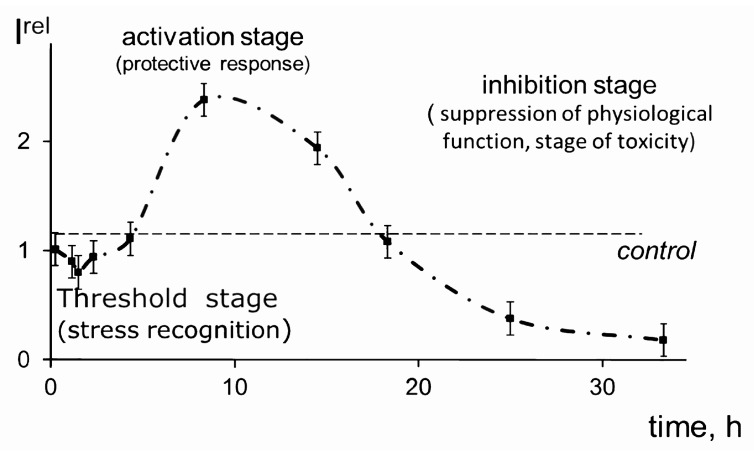
Bioluminescence kinetics of bacteria in a solution of americium-241, 3 kBq/L.

The bacterial bioluminescence kinetics in a solution of americium-241, an alpha-emitting radionuclide of high specific radioactivity, is presented in Figure 5.

**Figure 6:**
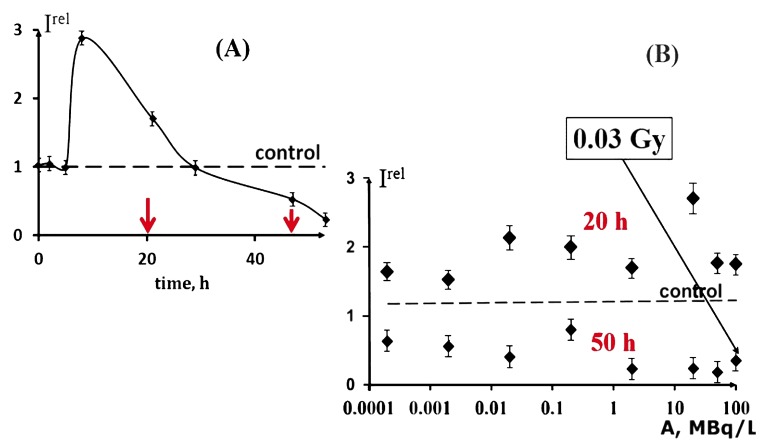
Effect of tritiated water on the bioluminescence of bacteria. (**A**) Bioluminescence kinetics of bacteria in tritiated water, 2 MBq/L; (**B**) bioluminescence intensity vs. activity concentration of tritiated water, A, at 20 and 50 h exposures.

Similar kinetic changes were obtained on exposure to the beta-emitting radionuclide tritium [22,61], Figure 6A reveals the same three stages in the bacterial luminescence response, with the activation stage included. The activation is a main peculiarity of interest in the response of the bacteria to low-dose radiation from alpha- and beta-emitting radionuclides (americium-241 and tritium, respectively). The responses are discussed in terms of “radiation hormesis”, as well as the “protective response of organisms”.

Additionally, an independence of the bioluminescence bacterial response from tritium activity concentration was found for the low-dose exposures [19,22,61]. To demonstrate this peculiarity, the time of exposure to tritium was fixed at 20 and 50 h, corresponding to the activation and inhibition stages of the bacterial exposure (Figure 6A). The bioluminescence intensity at different concentrations of tritium is presented in Figure 6B for 20 and 50 h exposure times. It can be seen here that the monotonic dependence is absent in a wide interval of activity for concentrations of tritium from 0.0001 to 200 MBq/L. This result can be explained in terms of the adaptation ability of the bacterial cells to low-dose radiation. It should be noted that the conventional limit of a low-dose interval (0.1 Gy) was not exceeded in the experiments.

Activation of the bacterial bioluminescence by tritium was demonstrated in a series of experiments. Bi-phasic dependence (activation and inhibition) was found in [22,61], and mono-phasic dependence (only activation) was found in [73,74].

To date, there are two hypothetical mechanisms that describe radiation hormesis; this phenomenon is associated with either DNA damage or membrane processes [63,66,67,75,76]. An original approach was applied in [73] to test the involvement of genetic changes in the activation of bacterial bioluminescence by tritium: using tritium-labeled films as a solid source of tritium radiation, the authors demonstrated that tritium activates bacterial luminescence without penetration into the cells. Additionally, no mutations were found in bacterial DNA (16S rRNA gene responsible for the vital functions of the bacterial cells [77]) under low-intensity irradiation of the alpha, beta, and gamma types [74]. A conclusion was made that the bioluminescence response is not associated with mutations in the tested gene. An alternative (non-genetic) mechanism of biological regulation which is related to the cell membrane processes, water media ionization, and formation of reactive oxygen species (ROS) under low-dose radiation exposures should be considered.

The biological role of ROS in toxic and adaptive effects is a challenge for modern molecular sciences, and mechanisms of ROS interaction with biological molecules are now being intensively studied [78,79,80].

Recent results [81] demonstrated that the exposure of marine bacteria to low-intensity irradiation from tritium increased the ROS content in the bacterial environment considerably, and a rise of the ROS content correlates with intensification of the bacterial bioluminescence intensity. These correlations were explained with a “trigger” function of products of tritium decay, signaling the role of ROS, and a “bystander effect” in the bacterial suspension.

Previously [60], the effect of americium-241 on luminous bacterium was attributed to ROS generated in aqueous solutions as secondary products of the radioactive decay. The effects of americium-241 and tritium on luminous bacteria were compared in [61] at comparable radiation doses; a higher impact of alpha irradiation from americium-241 was found. The result was related with the different energies of radioactive decay of americium-241 and tritium (5637.8 and 18.6 keV [82], respectively) and much higher ROS concentration in americium-241 water solutions as compared to in tritiated water. The authors discussed [60] a biological role of ROS generated in aqueous solutions at low-dose exposures.

Diffuse reflectance infrared Fourier transform (DRIFT) spectroscopic studies on *Photobacterium phosphoreum* biomass [83] showed that a low-dose alpha radioactivity effect of americium-241 was transmitted by live cells mainly to the bacterial bioluminescence enzyme system, with negligible structural or compositional changes in cellular macrocomponents. The effect of tritiated water on *P. phosphoreum* cells was also preliminarily studied using DRIFT spectroscopy in [84]. Under low-dose beta irradiation of tritium, some shifting of the amide I band of cellular proteins (sensitive to changes in the protein secondary structure) was found. Based on previous DRIFT spectroscopic results [85], the data were interpreted as a specific response of bacterial cells to stress induced by chronic low-dose radioactivity in tritiated water. It is noteworthy that similar DRIFT spectral changes in the amide I band were observed in rhizobacteria under exposures to other stress factors [85].

**Figure 7:**
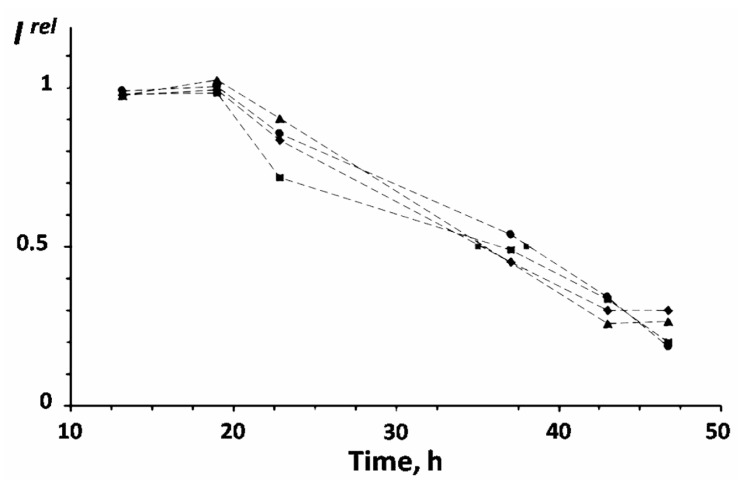
Kinetics of bioluminescence intensity (*I^rel^*) of *Photobacterium phosphoreum* under exposure to gamma radiation, ^137^ Cs, 20 °C. The error for *I^rel^* was 10%. (Dose rates: ■−4100; ◆−1040, •−460, ▲−150 µGy/h.

For the first time, the bioluminescence of bacteria was used to monitor the toxicity of gamma rays in [86]; the effects of average and high doses were under investigation. Exposure of the luminous bacteria to low-dose gamma radiation was studied in [72]. The dose–effect dependencies were of a stochastic nature; however, the dependencies of exposure time–effect were evident (Figure 7). Bioluminescence activation was not found under low-dose gamma radiation exposure; the bioluminescence kinetics corresponded to the threshold model (Figure 7), which was supposed as a particular case of the hormesis model [2,5]. Probably, the high energy and lower ionization ability of gamma rays (as compared with alpha and beta particles) are responsible for reducing the bacterial adaptive response. Additionally, an independence from dose rates was found for the low-dose gamma irradiation (Figure 7), similar to the low-dose effect of tritium (Figure 6B).

One more finding for gamma radiation effects was demonstrated in [72]: lowering the temperature (from 20 °C down to 10 °C and 5 °C) decreased the sensitivity of the bacterial cells to the low-dose gamma radiation; bioluminescence inhibition was not observed at 10 °C and 5 °C in contrast to at 20 °C. This result was generally explained by the temperature dependence of metabolic processes, including radiation-induced ones.

Hence, experiments with bacterial cells demonstrated an independence of their bioluminescence response on the intensity of irradiation (activity concentration for alpha/beta radionuclides americium-241/tritium and dose rate for gamma radiation) under low-dose exposures; however, time dependence was evident, corresponding to hormesis (for alpha/beta radionuclides americium-241/tritium) or threshold (for gamma radiation) models. “Cellular adaptive response” can be concerned with (1) independency of the response from irradiation intensity and (2) hormesis/threshold type of time response.

**Figure 8:**
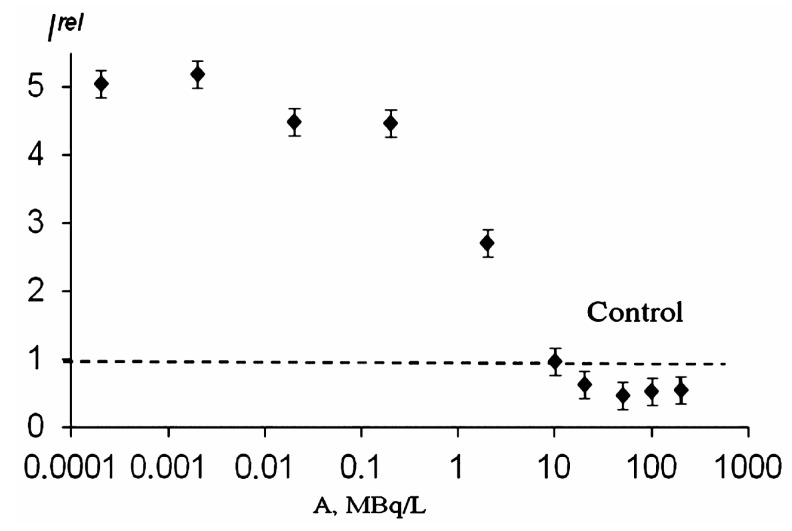
Bioluminescent intensity of an enzyme system, *I^rel^*, vs. the specific radioactivity of tritiated water, A.

The question is whether these two peculiarities are inherent in a biological system of a lower level of organization—enzymatic reactions. Effects of alpha- and beta-emitting radionuclides (americium-241 and tritium) on the bioluminescence system of coupled enzyme reactions catalyzed by bacterial luciferase and NADH: FMN oxidoreductase were studied in [21,22]. Bioluminescence activation and inhibition were observed. A monotonic dependence on the concentration of radionuclides was also found. The example of this dependence is shown in Figure 8.

**Figure 9:**
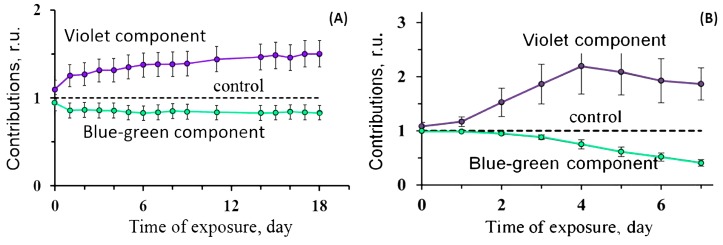
Relative contributions of spectral components to the fluorescence spectra of CLM-containing protein (“discharged obelin” from *Obelia longissima)* exposed to (**A**) tritiated water, 200 MBq/L [87]; or (**B**) gamma radiation, ^137^ Cs, 2 mGy/h, 20 °C.

The simplest luminescent bioassay based on CLM-containing protein (“discharged obelin” from *Obelia longissima*) did not demonstrate an activation stage under low-intensity exposures. Figure 9 presents the time courses of colored fluorescence contributions under exposure to beta (A) and gamma (B) irradiation according to [87,88]. The increase in violet contributions (as compared to the non-irradiated control) in both cases is evidence of proton transfer inhibition in the excited CLM–apoprotein complex. As suggested in Section 2, this effect can be accounted for by inactivation of the protein.

Dependence of the response of CLM-containing protein on the temperature was demonstrated [88] for low-intensity gamma radiation, as well.

Hence, the simplest enzymatic bioassay systems (based on enzymatic chemiluminescent reactions and physicochemical processes in the protein complex) show a lower ability for adaptive response to low-dose radiation. Bioluminescence activation was found in the bacterial enzymatic assay. However, in contrast to the bacterial cells, it demonstrated a dependence on the intensity of irradiation. Fluorescent-protein-based bioassay did not show any activation stage in the course of chronic exposure to radiation of the beta and gamma types. However, the lack of experimental results does not allow for making definite conclusions. These studies should be further developed.

## 4. Low-Concentration Effects of Bioactive Compounds: Antioxidant Activity via Bioluminescence Bioassays

Investigation of the low-concentration effects of bioactive compounds has been developed since the 1960s [63,89,90,91]. The hormesis concept has progressed to describe the intensification of physiological functions of organisms under low-concentration exposures [2,3,4,90,91,92,93].

Bioluminescence bioassays based on luminous marine bacteria and their enzymes are excellent tools to study the low-concentration effects due to their features mentioned before:

1. High rates of test procedure, which
  - provide statistical reliability of the bioassay results and
  - can exclude the genetic level of analysis, appealing to biochemical, chemical, and physicochemical processes in cells;
2. The possibility to compare effects of bioactive compounds at different organization levels—cellular and enzymatic.

**Figure 10:**
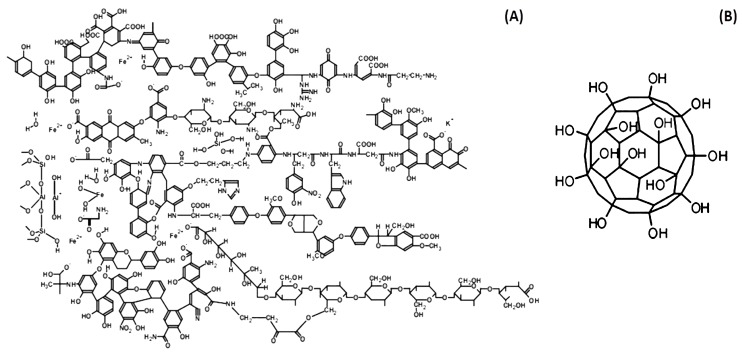
Hypothetical structure of bioactive compounds: (**A**) fragment of humic substances, (**B**) fullerenol C-60.

The bioassays based on luminous marine bacteria and bacterial enzymes were used to study the antioxidant properties of bioactive compounds of natural and artificial origination. *HS* and *F*s were chosen as examples of these compounds. The *HS* are products of natural oxidative transformation of organic matter in soils and sediments, attenuators of toxicity in natural water bodies and soils. *F*s are specific allotropic forms of carbon, nanosized polyhydroxylated water-soluble derivatives of fullerenes, bioactive compounds, and perspective pharmaceutical agents. Hypothetical structures of *HS* [94] and *F* are presented in Figure 10. The antioxidant activity of *HS* was studied in [12,95,96,97,98] and that of *F*s in [98,99,100,101].

Cellular and enzymatic assays were used to evaluate the general toxicity (GT) under conditions of oxidative stress; this stress was modeled in the solutions of exogenous organic or inorganic oxidizers (1,4-benzoquinone or potassium ferricianide, respectively). Additionally, an enzymatic bioassay was shown to be specific to oxidizers [31,102]; it can be used to monitor the oxidative toxicity (OxT) of the solutions. This toxicity type characterizes the redox properties of toxic compounds, while the other type of toxicity, GT, mentioned before, integrates all the interactions of toxic compounds with a bioassay system: redox reactions, non-polar and polar interactions, and so on. The GT is concerned with suppression of the maximal bioluminescence intensity, while the OxT uses a specific kinetic parameter: the induction bioluminescence period. The latter appears in the presence of exogenous oxidizers depending on their redox potential and concentration [31]. Justification for GT and OxT application to evaluate the toxicity of bioactive compounds was presented in [12,95,96,97,98,99,100,101].

**Figure 11:**
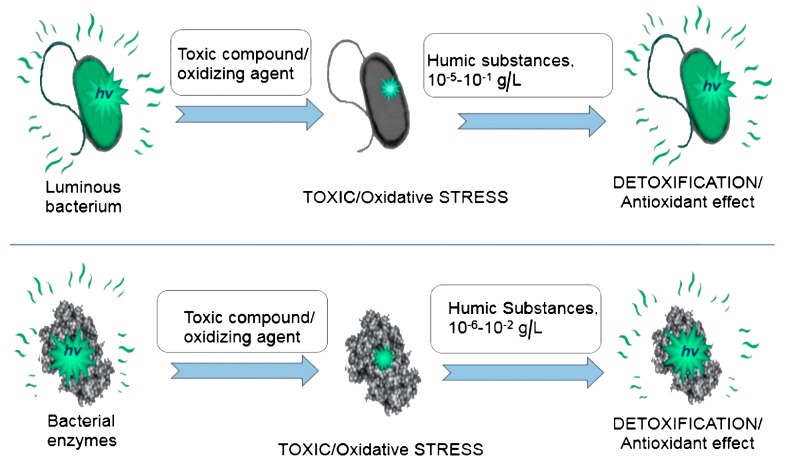
Principle of antioxidant efficiency evaluation using bacteria-based (upper path) and enzyme-based (lower path) bioluminescent assays.

The principle of antioxidant efficiency evaluation is presented in Figure 11 for bacteria-based (upper line) and enzyme-based (lower line) bioassays; humic substances were chosen here as an example of an antioxidant agent.

The antioxidant efficiency of bioactive compounds is characterized by antioxidant coefficients *D_GT_* or *D_OxT_*, corresponding to GT or OxT monitoring. Values of *D_GT_* of >1 or *D_OxT_* of >1 reveal antioxidant effects of the bioactive compounds.

**Figure 12:**
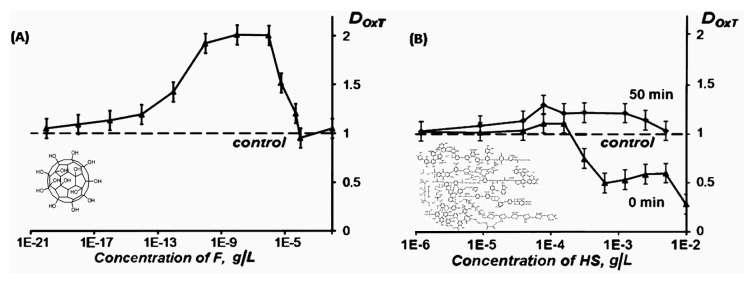
Antioxidant coefficients *D_OxT_* of *F* (**A**) and *HS* (**B**) in a model solution of organic oxidizer (1,4-benziquinone) [100]. The time of incubation of *HS* with the oxidizer (0 min and 50 min) is indicated in (B). Enzymatic assay.

Paper [100] compared the biological activity of carbon nano-structures of natural and artificial origination, i.e., *HS* and *F*. The representative of the group of *F*s, C_60_O_y_(OH)_x_ where *y* + *x* = 20–22, was chosen. Enzymatic assay was used to monitor the toxicity and antioxidant activity of the bioactive compounds. Toxic concentrations of *HS* and *F* inhibiting bioluminescence were determined as >0.002 g/L and >0.01 g/L, respectively. Bacterial bioluminescent assay demonstrated the following toxic intervals: >0.1 g/L and >0.01 g/L for *HS* [96] and *F* [101], respectively. All toxic concentrations were excluded from the further antioxidant experiments. The antioxidant coefficients of the bioactive compounds and ranges of their active concentrations were determined in solutions of model oxidizers. Figure 12 presents an example of the dependencies of antioxidant coefficient *D_OxT_* on the concentrations of *F* (A) and *HS* (B).

Both *HS* and *F* demonstrated low-concentration antioxidant activity (*D_OxT_* > 1). However, their quantitative antioxidant characteristics were different: The *D_OxT_* values of *F* were higher and its antioxidant activity covered a wider concentration range, as seen from Figure 12. The antioxidant activity of *HS* was found to be time dependent (Figure 12B), while the *F* antioxidant effect showed independency from time. The *HS* antioxidant effect did not depend on the amphiphilic characteristics of the environment (*D_OxT_* values were 1.3 in solutions of inorganic and organic oxidizers), while the *D_OxT_* of *F* was maximal in solutions of the organic oxidizer (*D_OxT_* = 2.0). This difference in the effects of the bioactive compounds in solutions of organic and inorganic oxidizers may be concerned with their hydrophobic interactions in enzymes or cellular membranes. Changes in fluidity and the structural organization of lipid bilayers in hydrophobic fragments of membranes by *F* were previously reported in [103].

In [100], the differences in the toxic and antioxidant effects of *F* and *HS* were attributed to the structure of these compounds. The non-rigidity of humic macromolecules determines their diffusion restrictions, which result in higher toxicity and time dependence of their antioxidant coefficients. The ability to decrease the ROS content in water solutions probably contributes to the higher toxicity of *HS*, as well. Non-rigidity and polyfunctionality may be responsible for the unification of *HS* properties in solutions of oxidizers of different hydrophilic/hydrophobic characteristics. The low-concentration antioxidant activity was explained by the catalytic redox activity of π-fragments of the bioactive structures.

The antioxidant property of highly diluted solutions of fullerenols was attributed [99] to a hormesis phenomenon. The bacteria-based and enzyme-based assays demonstrated similar peculiarities of the antioxidant processes: (1) ultralow concentrations of *F*s are active (ca. 10^–17^–10^−4^ and 10^–17^–10^−5^ g/L, respectively), (2) monotonic dependence of the antioxidant coefficients on *F* concentrations was not observed, and (3) detoxification of solutions of the organic oxidizer was more effective than that of the inorganic one. The antioxidant properties were concerned with the adaptive cellular response under low-dose exposures. Sequence analysis of 16S ribosomal RNA was carried out for longer exposures, and mutations in bacterial DNA were not revealed.

The biological efficiency of low and ultralow concentrations of hydrated fullerenes was studied and discussed earlier in [104,105]. The effect was attributed to the fullerenes’ ability to regulate a dynamic structure and to adjust redox processes in aqueous systems. Earlier [106], a role of aqueous media in fullerenols’ antiradical activity was discussed. Probably, ROS in aqueous media are able to contribute to the antioxidant effect of fullerenols.

In [98,101], the antioxidant activity of *F*s was related with modifications of their surface by oxygen substituents, as well as the presence of exo- or endohedral metal atoms. Differences in the antioxidant activity of these *F*s were explained through their electron donor/acceptor properties and differences in catalytic activity.

As an outlook,

1. Bioactive compounds can produce toxic (inhibition) and antioxidant (activation) effects. The toxic effect of *HS* was found at higher concentrations (>0.1 g/L for bacterial assay and >0.01 g/L for enzymatic assay), while the antioxidant effect of *HS* was found at lower concentrations. Similar to *HS*, all *F*s showed toxic effects at higher concentrations of >0.01 g/L, while their antioxidant effect was found at low and ultralow concentrations: ca. 10^−17^–10^−4^ and 10^−17^–10^−5^ g/L for bacteria-based and enzyme-based assays, respectively. The range of active *F* concentrations might correspond to several tens of molecules per liter.

2. No monotonic dependencies of the antioxidant coefficients on the concentration of the bioactive compounds were found in a wide concentration range (up to 15–17 orders for *F*s).

3. Both bacterial and enzymatic assays demonstrated an antioxidant effect of the bioactive compounds. This result reveals a role of biochemical processes in the low-concentration antioxidant effects of bioactive compounds.

4. Cellular and enzymatic bioluminescent assays showed that detoxification of solutions of the organic oxidizer was more effective than that of the inorganic oxidizer, with this indicating the importance of hydrophobic interactions in the antioxidant mechanism.

Hence, similar to low-dose radiation, the antioxidant effects of bioactive compounds (*F*s and *HS*) showed (a) a positive response of luminous bacteria and their enzymes to low-concentration exposures and (b) an absence of linear concentration–effect dependencies.

## 5. Conclusions

Luminescence bioassay systems can provide a non-genetic approach to low-intensity exposures due to high rates of registration of the luminescence response. Study of the biochemical, chemical, and physicochemical (polar, apolar, hydrophobic) processes using luminescence signaling can develop the “exposomic” concept [34,35,36,37].

The biochemical, chemical, and physicochemical processes have not, until now, attracted much attention in the context of the concept of hormesis. Application of simple model organisms and biochemical systems provides exposure sciences with fundamental support. Compensatory effects can be strictly studied in terms of chemical equilibrium by analyzing the individual equilibrium constants and concentrations in complex systems of coupled chemical reactions.

The results presented in this review reveal a dependence of biological responses on time of exposure and their independence from the intensity of the exposure. Attention was also paid to the uncertainty of dose–response relations. These features of low-dose exposures should be analyzed in detail at the molecular level in further studies.

## Abbreviations

CLM	Coelenteramide
DRIFT	Diffuse reflectance infrared Fourier transform
F	Fullerenol
FMN	Flavin mononucleotide
GT	General toxicity
HS	Humic substances
NADH	Nicotinamide adenine dinucleotide
OxT	Oxidative toxicity
ROS	Reactive oxygen species
